# p53 and p63 Proteoforms Derived from Alternative Splicing Possess Differential Seroreactivity in Colorectal Cancer with Distinct Diagnostic Ability from the Canonical Proteins

**DOI:** 10.3390/cancers15072102

**Published:** 2023-03-31

**Authors:** Ana Montero-Calle, María Garranzo-Asensio, Rebeca M. Torrente-Rodríguez, Víctor Ruiz-Valdepeñas Montiel, Carmen Poves, Jana Dziaková, Rodrigo Sanz, Cristina Díaz del Arco, José Manuel Pingarrón, María Jesús Fernández-Aceñero, Susana Campuzano, Rodrigo Barderas

**Affiliations:** 1 Chronic Disease Programme (UFIEC), Instituto de Salud Carlos III, 28220 Madrid, Spain; ana.monteroc@isciii.es (A.M.-C.); mgarranzo@isciii.es (M.G.-A.); 2 Department of Analytical Chemistry, Faculty of Chemistry, Complutense University of Madrid, 28014 Madrid, Spain; rebecamt@ucm.es (R.M.T.-R.); vrvmontiel@ucm.es (V.R.-V.M.); pingarro@quim.ucm.es (J.M.P.); susanacr@quim.ucm.es (S.C.); 3 Gastroenterology Unit, Hospital Universitario Clínico San Carlos, 28040 Madrid, Spain; carmen.poves@salud.madrid.org; 4 Surgical Digestive Department, Hospital Universitario Clínico San Carlos, 28040 Madrid, Spain; 5 Surgical Pathology Department, Hospital Universitario Clínico San Carlos, 28040 Madrid, Spainjmariajesus.fernandez@salud.madrid.org (M.J.F.-A.)

**Keywords:** colorectal cancer, immunomics, autoantibodies, p53 family, p53 and p63, alternative splicing, proteoform, POC-like device, biosensor, humoral immune response, diagnosis

## Abstract

**Simple Summary:**

The humoral immune response in cancer has been demonstrated to be useful for distinguishing patients from healthy individuals using serum or plasma. Our study aimed to assess whether p53 and p63 proteoforms derived from alternative splicing could have a differential seroreactivity and higher diagnostic value than canonical proteins in colorectal cancer. Using luminescence assays and biosensing approaches with the proteoforms expressed in vitro fused to HaloTag, we demonstrate the appearance of a differential seroreactivity among the proteoforms in colorectal cancer patients. Our findings reveal increased complexity of the humoral immune response in cancer against specific autoantigens, since specific seroreactivity and different diagnostic values were observed among the p53 and p63 proteoforms and the canonical proteins.

**Abstract:**

Colorectal cancer (CRC) is the third most common cancer and the second most frequent cause of cancer-related death worldwide. The detection in plasma samples of autoantibodies against specific tumor-associated antigens has been demonstrated to be useful for the early diagnosis of CRC by liquid biopsy. However, new studies related to the humoral immune response in cancer are needed to enable blood-based diagnosis of the disease. Here, our aim was to characterize the humoral immune response associated with the different p53 and p63 proteoforms derived from alternative splicing and previously described as aberrantly expressed in CRC. Thus, here we investigated the diagnostic ability of the twelve p53 proteoforms and the eight p63 proteoforms described to date, and their specific N-terminal and C-terminal end peptides, by means of luminescence HaloTag beads immunoassays. Full-length proteoforms or specific peptides were cloned as HaloTag fusion proteins and their seroreactivity analyzed using plasma from CRC patients at stages I-IV (*n* = 31), individuals with premalignant lesions (*n* = 31), and healthy individuals (*n* = 48). p53γ, Δ40p53β, Δ40p53γ, Δ133p53γ, Δ160p53γ, TAp63α, TAp63δ, ΔNp63α, and ΔNp63δ, together with the specific C-terminal end α and δ p63 peptides, were found to be more seroreactive against plasma from CRC patients and/or individuals with premalignant lesions than from healthy individuals. In addition, ROC (receiver operating characteristic) curves revealed a high diagnostic ability of those p53 and p63 proteoforms to detect CRC and premalignant individuals (AUC higher than 85%). Finally, electrochemical biosensing platforms were employed in POC-like devices to investigate their usefulness for CRC detection using selected p53 and p63 proteoforms. Our results demonstrate not only the potential of these biosensors for the simultaneous analysis of proteoforms’ seroreactivity, but also their convenience and versatility for the clinical detection of CRC by liquid biopsy. In conclusion, we here show that p53 and p63 proteoforms possess differential seroreactivity in CRC patients in comparison to controls, distinctive from canonical proteins, which should improve the diagnostic panels for obtaining a blood-based biomarker signature for CRC detection.

## 1. Introduction

Alternative splicing, a mechanism of posttranscriptional mRNA processing, is an important regulatory process whereby several mature transcript variants are produced from a single gene. Therefore, alternative splice variants of one gene encode multiple protein variants with diverse physiological functions. Estimations indicate that around 92–95% of human multiexon genes undergo alternative splicing [1,2]. Abnormal alterations of splicing, such as cis- or trans-factor alterations, may interfere with normal cellular homeostasis, leading to cancer development and/or progression [3,4,5,6].

p53, p63, and p73 transcription factors compose the p53 protein family. Similar domain structures and amino acid sequence homology are found in the transactivation (TAD), DNA-binding (DBD), and oligomerization (OD) domains of the three proteins [7]. As assessed by phylogenetic studies, the p53 family derived from an ancestral p63/73-like gene [8,9]. The function of this ancestral gene consisted of maintaining the genetic stability of germ cells [10]. Collectively, p53, p63, and p73 control important biological processes, such as cell differentiation, proliferation, cell death, and apoptosis. Additionally, they have been shown to possess different important biological roles, with the three members of the p53 family acquiring unique functional specificities since their duplication and divergence during evolution [11,12]. In this sense, it has been shown that the dysregulation of different proteoforms of the p53 family is crucial in tumorigenesis and importantly affects responses to tumor therapy.

**Figure 1:**
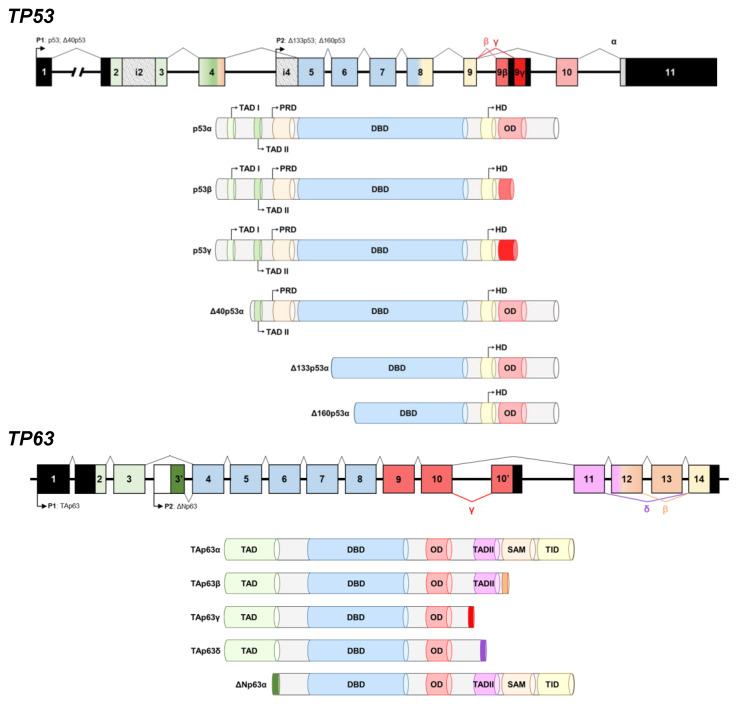
Schematic representation of the domains of described human p53 and p63 proteoforms derived from the alternative splicing of *TP53* and *TP63* genes. Alternative promoters (P1 and P2) are indicated. Alternative splicing events at the C-terminal generate variants α, β, and γ. Exon skipping or premature transcription termination produces variant δ. Truncated transactivating domains of p53 produce Δ40, Δ133, and Δ163 p53 proteoforms, whereas truncation of transactivation domains in p63 produce ΔN proteoforms. Transactivation domains (TAD), DNA-binding domain (DBD), proline-rich domain (PRD), sterile alpha motif domain (SAM), C-terminal inhibitory domain (TID), oligomerization domain (OD), and hinge domain (HD) are depicted.

Preservation of genome integrity by preventing the proliferation of stressed cells (more likely to mutate and grow aberrantly), thus, preventing cancer formation, is the best-known function of p53, [13,14]. Indeed, the importance of p53 can be illustrated because of p53 activity loss is a common hallmark of cancer, occurring through protein inactivation or gene mutation [15,16]. In normal conditions, *TP53* gene transcription can be produced from two different promoters (P1 and P2). P1 is located upstream of exon-1, whereas P2 is located internally in intron-4. The alternative P2 promoter leads to an NH_2_-truncated p53 protein termed Δ133p53, which starts at codon 133. Additionally, exon-9 can suffer from alternative splicing to produce p53, p53β, and p53γ proteoforms, with p53β and p53γ lacking the oligomerization domain. Therefore, the human p53 gene can encode different mRNAs codifying for twelve proteoforms of p53: p53α (canonical), p53β, p53γ, Δ133p53α, Δ133p53β, and Δ133p53γ. Additionally, Δ40p53α, Δ40p53β, and Δ40p53γ are generated from P1 if intron-2 is retained in the transcript and due to alternative splicing of exon-9, whereas Δ160p53α, Δ160p53β, and Δ160p53γ are synthesized due to alternative splicing of exon-9 and the use of the alternative promoter in intron-4, starting from the Met at codon 160 [7,17,18]. Thus, Δ40p53 proteoforms lack the transactivation domain I (TADI), whereas Δ133p53 and Δ160p53 lack both transactivation domains (TADI and TADII) (Figure 1).

p63 and p73, the two homologs of the tumor suppressor transcription factor p53, can bind and activate most of the p53-responsive promoters, due to their high structural similarity. Therefore, although there exist overlapping functions among the p53 family members as induction of apoptosis in response to cellular stress, and p63 and p73 cooperate with p53 in the absence of stress in the regulation of tumorigenesis, the extensive structural variability within the p53 family determined by their tight splicing regulation provides unique physiological roles to p63 and p73 proteins. In this sense, it has been reported that p63 is involved in squamous epithelia development. On the other hand, p73 is needed for development of the nervous and olfactory systems, while p73 is essential for neuronal differentiation. In this regard, analyses of p63- and p73-deficient mice showed important malformation defects at birth, or the absence of skin, hair, mammary, lachrymal, and salivary glands [19,20,21,22], and complex defects in neuronal development [21,23], respectively. As a result, protein imbalance in the p53 family produces a significant proportion of congenital developmental abnormalities in humans. Different proteoforms have been also described for p63. The *TP63* gene possess two distinct sites for transcription initiation, upstream of exon-1 (P1) and from an internal promoter located in intron-3 (P2), resulting in the ΔNp63 proteoforms without the TAD domain. In addition, alternative splicing at the 3′ end of the RNA produces the different, shorter p63 C-terminal proteoforms. Thus, up to four different C-terminal proteoforms caused by alternative splicing and which lack the transactivation inhibitory domain (TID) have been described for p63 (α, β, γ and δ), with γ and δ also lacking the sterile alpha motif (SAM) (Figure 1).

In cancer, the p53-family is aberrantly expressed, with p63 and p73 showing low mutational levels in contrast to p53 that is extensively mutated in all cancer types [12,24,25,26,27]. In particular, ΔNp63 and ΔNp73 proteoforms are frequently overexpressed in a wide range of tumors, where they are associated with poor prognosis [12,27]. Interestingly, these aberrant alterations (overexpression, point mutation, frameshifts, etc.) are major causes of induction of a humoral immune response in cancer patients [28]. In this sense, p53 autoantibodies are considered one of the best cancer biomarkers because of their specificity for detecting major solid tumors at early cancer stages [28,29,30,31]. This has led p53 to be proposed as the main cancer autoantigen that should be included in any blood-based cancer diagnostic test [28,29,30,31]. However, little is known about the roles of p63 and p73 autoantibodies in cancer, the differential seroreactivity of the multiple proteoforms of the p53-family, or whether they could also have a differential diagnostic ability compared with the canonical proteins. Recently, we reported that ΔNp73 proteoforms of p73 showed higher specific seroreactivity and a greater ability to discriminate colorectal cancer (CRC) patients from controls compared with the canonical p73 protein [32]. This was especially evident for the discrimination between colorectal premalignant individuals and controls [32]. As these findings might be extended to p53 and p63 proteoforms, having relevance and an important impact on cancer prevention by predicting premalignant colorectal tumors, we aim here to focus on the analysis of the differential seroreactivity against the known proteoforms of p53 and p63, to determine the existence of cryptic epitopes absent in the canonical proteins, and whether the proteoforms could have higher diagnostic ability than the reference proteins.

**Figure 2:**
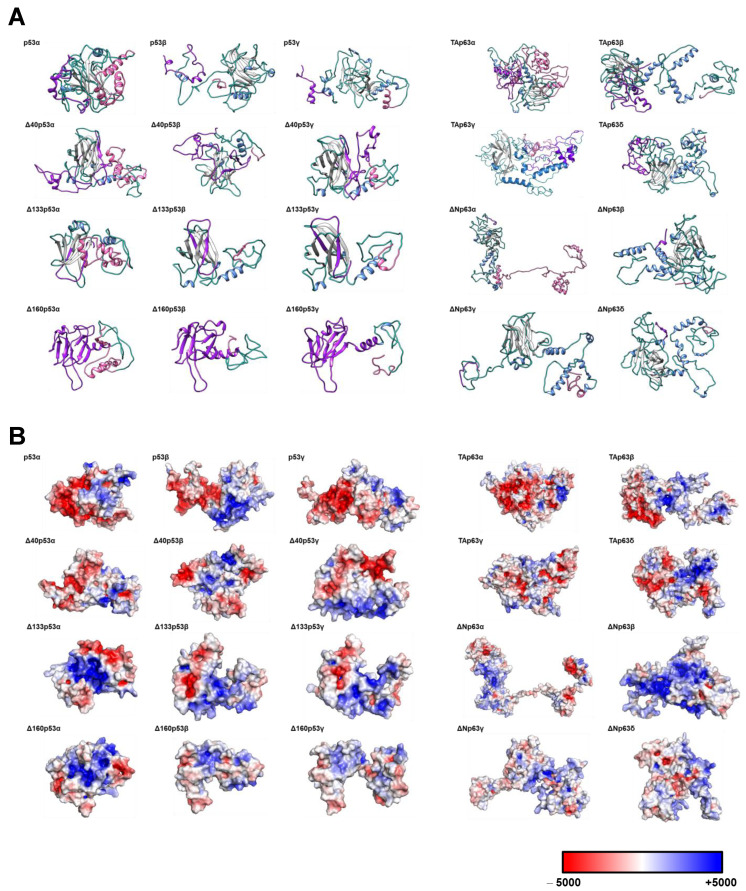
Prediction of the 3D structures of p53 and p63 proteoforms. (**A**) Prediction of the 3D structures of the p53 and p63 proteoforms. (**B**) Prediction of the electrostatic potential of the p53 and p63 proteoforms. Electropositive and electronegative charged regions are colored in blue and red, respectively. Neutral regions are colored in white.

Prior to starting the study and cloning and expressing all proteoforms for investigating differential seroreactivity associated with the potential existence of cryptic epitopes, we obtained 3D models to assess potential structural differences of p53 and p63 proteoforms (Figure 2). The 3D models showed not only that the differences in the amino acid sequences of p53 and p63 proteoforms produced remarkable changes in the 3D structure (Figure 2A) and electrostatic surface potential (Figure 2B), but also indicated more differences at the N- and C-terminal ends of the proteoforms in comparison with the canonical proteins. Thus, these 3D models suggest that p53 and p63 proteoforms could contain specific epitopes of cancer autoantibodies absent in the canonical proteins, supporting the interest in their analysis to elucidate whether they have differential seroreactivity to that of canonical proteins and higher diagnostic ability for CRC. To this end, we cloned and expressed in vitro all p53 and p63 proteoforms and specific N- and C-terminal peptides fused to HaloTag (Figure 1). We analyzed their seroreactivity by HaloTag luminescence assays using a cohort of 110 plasma samples from CRC patients, premalignant individuals, and healthy controls. Finally, we constructed a specific biosensing POC-like platform for the simultaneous analysis of selected proteoforms with the highest diagnostic ability, for its potential implementation with clinical purposes.

## 2. Materials and Methods

### 2.1. Plasma of Patients and Controls

This study on biomarker discovery was approved by the Ethical Review Boards of the Instituto de Salud Carlos III and Hospital Universitario Clínico San Carlos (CEI PI 13_2020-v2). Plasma samples were obtained from the IdISSC biobank of the Hospital Clínico San Carlos after Ethical Committee approval. Written informed consent was obtained from all participants. All experiments were performed in agreement with relevant guidelines and regulations.

A panel of 110 plasma samples from CRC patients (*n* = 31), individuals with premalignant lesions (*n* = 31; low- or high-grade colorectal adenomas), and healthy control individuals (*n* = 48; healthy asymptomatic individuals, FOBT-positive and colonoscopy-negative individuals) was used for seroreactivity analyses of the humoral immune response against p53 and p63 proteoforms in comparison with the canonical proteins (Table 1 and Table S1). Plasma samples were collected using a standardized sample collection protocol and stored at −80 °C until use [32,33].

### 2.2. In Silico Modeling of the Proteins

Structural models in PDB format were generated using the I-TASSER online server (https://zhanggroup.org/I-TASSER/ (accessed on 1 January 2021)). Three-dimensional models were obtained with PyMOL (Schrödinger, LCC, New York, NY, USA). The PyMOL adaptive Poisson Boltzmann solver (APBS) electrostatics plugin was used for the prediction of the electrostatic surface potential of the indicated proteoforms.

### 2.3. Gateway Plasmid Construction, Gene Cloning, DNA Preparation and Protein Expression

TP53 and TP63 cDNAs in pDONR221 vectors were obtained from the DNASU Plasmid Repository (https://dnasu.org/DNASU/ (accessed on 21 April 2013)). Verified TP53 and TP63 sequence plasmids were directly used as DNA templates for subsequent cloning of full-length and specific peptides encoding cDNA. The different p53 and p63 proteoforms encoding cDNA were PCR amplified with specific oligonucleotides containing the attB ends from the corresponding pDONR221 vectors (Table S2). PCR products were purified by precipitation with 100% ethanol and directly cloned in pDONR221 entry vector by a BP clonase reaction (Invitrogen, Carlsbad, CA, USA) according to the manufacturer instructions [31]. The ORFs were transferred by LR clonase reactions (Invitrogen, Carlsbad, CA, USA) to a pANT7_cHalo expression vector, developed in the laboratory, for in vitro protein expression [30]. Alternatively, differential N-terminal and C-terminal end peptides specific to each p53 and p63 proteoform were obtained by PCR using specific oligonucleotides (Table S2), purified by precipitation with 100% ethanol, cloned in the pDONR221 entry vector by a BP clonase reaction (Invitrogen, Carlsbad, CA, USA), and transferred by LR clonase reactions (Invitrogen, Carlsbad, CA, USA) to a pANT7_cHalo or pJFT7_nHalo expression vector, developed in the laboratory, for in vitro protein expression. N-terminal and C-terminal end specific peptides encoding cDNA were cloned as N-terminal and C-terminal fused proteins, respectively. Sequences of p53 and p63 proteoforms and specific peptides are summarized in Table S3.

To obtain high-quality DNA for in vitro protein expression, plasmids transformed onto TOP10 *E. coli* cells were grown in 250 mL Luria Bertani (LB) supplemented with the appropriate antibiotic (100 μg/mL for ampicillin and 40 μg/mL for kanamycin). Donor and expression plasmid DNAs were purified by miniprep (Neobiotech, Pasadena, CA, USA) and verified by agarose gel electrophoresis and sequencing prior to subsequent use (Figure S1).

To carry out the immunoassays and their analysis in the biosensing platforms, the 20 constructions of p53 and p63 proteoforms and the 13 specific peptides were expressed in vitro using the 1-Step Human Coupled IVT Kit HeLa cell lysate DNA (Thermo Fisher Scientific, Waltham, MA, USA).

### 2.4. SDS-PAGE and Western Blot Analysis

SDS-PAGE and Western blot (WB) analysis to assess protein quality were performed using 0.67 μL of the in vitro expressed (IVT) protein extracts and optimized dilutions of specific monoclonal antibodies against p53, p63, or HaloTag, and their corresponding HRP-conjugated secondary antibodies (Table S4) [33,34,35]. Chemiluminescence signals were developed using the ECL Western blotting substrate (Thermo Fisher Scientific, Waltham, MA, USA). Signal detection was performed on an Amersham Imager 680 (GE Healthcare, Chicago, IL, USA).

### 2.5. Seroreactivity luminescence and Biosensing Assays

The seroreactivity of each of the 110 plasma samples (Table 1 and Table S1) at 1/400 dilution against p53 and p63 proteoforms and specific peptides was analyzed by luminescence assays using Magne HaloTag beads (MBs, Promega, Madison, WI, USA), as previously described [33,35,36,37]. An HRP-labeled anti-human IgG secondary antibody (Dako, Santa Clara, CA, USA) diluted 1/3000 was used for detection of autoantibodies specifically bound to the different proteoforms. As controls, the MBs covalently immobilized with HaloTag fusion proteoforms were detected with an anti-HaloTag mAb diluted 1/1000, and an HRP-conjugated anti-mouse IgG (Sigma Aldrich, Sofia, Bulgaria) diluted 1/1000. The signal was developed with the ECL Pico Plus chemiluminescent reagent (Thermo Fisher Scientific, Waltham, MA, USA) and recorded onto a Spark multimode microplate reader (Tecan Trading AG, Männedorf, Switzerland).

Alternatively, indicated proteoforms were analyzed by means of electrochemical biosensing assays using previously optimized protocols, using 2 µL of protein extract and 0.5 µL of MBs suspension per reaction [30,36,37]. As control to verify correct protein immobilization, HaloTag fusion proteins were detected with an anti-HaloTag mAb diluted 1/400, and an HRP-conjugated anti-mouse IgG (Sigma Aldrich, Sofia, Bulgaria) diluted 1/1000. The amperometric signal was developed in the presence of the hydroquinone (HQ)/H_2_O_2_ system, using disposable screen-printed carbon electrodes upon magnetic capture on the working electrode of MBs bearing the immunocomplexes [30,36,37].

### 2.6. Statistical Analysis

Microsoft Office Excel, GraphPad Prism 5, and the R program (v4.1.1) were used for the statistical analysis. Mean and standard error of the mean (SEM) were obtained with Microsoft Office Excel and GraphPad Prism 5. Statistical differences in the means of healthy individuals, premalignant individuals, and CRC groups from luminescence and biosensing datasets were assessed by means of non-parametric Mann–Whitney U testing. *p* values < 0.05 were considered statistically significant. Individual autoantibodies against each indicated proteoform were evaluated as plasma markers in premalignant individuals, CRC patients, and control individuals, by ROC curve using the R program (version 3.2.3) with the “Epi”, “ModelGood”, and “pROC” R packages to calculate the corresponding AUC (area under the curve), the maximized sensitivity and specificity values, and the threshold (cut-off value) for all indicated comparisons.

### 2.7. Data Availability

All data generated or analyzed during this study are included in the manuscript and its Supplementary Information Files.

## 3. Results

The canonical proteins of the three members of the p53 family (p53α, TAp63α, and TAp73α) have been analyzed as autoantibody targets in different cancer types. In addition, seroreactivity to the cancer autoantigen p53 has also been evaluated by using the most frequent p53 point mutants found in cancer and different N-terminal and C-terminal deletions of the protein, with the aim of identifying cryptic or masked epitopes [31]. Moreover, a recent analysis of several p73 proteoforms demonstrated the presence of a specific seroreactivity to ΔNp73 proteoforms, different to the seroreactivity of the canonical p73 protein in CRC. Additionally, it was also noticed that the diagnostic ability of ΔNp73α seroreactivity was also higher than that of seroreactivity to the canonical protein and other p73-derived proteoforms. Furthermore, the diagnostic effectiveness was improved by combining ΔNp73 and p73 seroreactivity for the detection of either CRC patients or premalignant individuals among healthy individuals. This study suggests that the different proteoforms of p53 and p63 could not only contain cryptic epitopes as p73 proteoforms -as 3D-models suggested (Figure 2), but might also produce a differential seroreactivity able to improve the diagnostic effectiveness of these cancer autoantigens [32].

Here, we have focused on addressing these questions by means of seroreactivity luminescence and biosensing assays. To this end, we cloned and expressed in vitro the different proteoforms of p53 and p63 fused to HaloTag at its C-terminal end, and the different specific N-terminal and C-terminal peptides of each p53 and p63 proteoform fused to HaloTag at its C-terminal or N-terminal end, to analyze their seroreactivity using a cohort of 110 plasma samples from CRC patients, individuals with premalignant lesions (low- and high-grade adenomas), and healthy asymptomatic individuals -FOBT-positive and colonoscopy-negative individuals- (Figure 1 and Figure S1).

### 3.1. Cloning, In Vitro Protein Expression of Fusion Proteins, and In Silico Analysis of the Proteoforms

**Figure 3:**
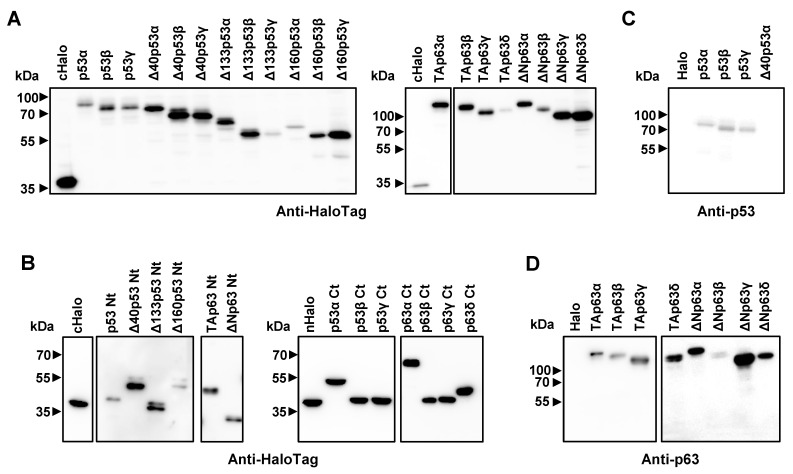
Cloning, and in vitro protein expression of the p53 and p63 proteoforms used as HaloTag fusion proteins in subsequent experiments. (**A**) Confirmation of the in vitro protein expression of full-length p53 and p63 proteoforms derived from alternative splicing as HaloTag fusion proteins was assessed by WB using an anti-HaloTag mAb. (**B**) Confirmation of the in vitro protein expression of the indicated specific p53 and p63 peptides derived from alternative splicing as HaloTag fusion proteins was assessed by WB using an anti-HaloTag mAb. (**C**) Immunostaining analysis using an anti-p53 mAb against the N-terminal end of unmodified p53 proteoforms confirmed their correct expression in vitro. (**D**) Immunostaining analysis using an anti-p63 mAb against a N-terminal peptide present in all p63 proteoforms confirmed their correct expression in vitro. The original western blot figures could be found in Figure S2.

The major bottleneck for the development of immunoassays is associated with protein expression and purification of stable and integral proteins with good yield and purity. Therefore, for their functional analyses, here we took advantage of cell-free systems for the timely production of p53 and p63 proteoforms and their specific N- and C-terminal peptides for direct use for seroreactivity analysis, instead of expressing them in heterologous systems (Figure 3).

For the development of our approach, we first cloned p53 and p63 proteoforms and specific N-terminal and C-terminal peptides in pDONR221 vector and transferred them to pANT7_cHalo or pJFT7_nHalo expression vectors by BP and LR clonase reactions for subsequent in vitro protein expression of the corresponding HaloTag fusion proteins (Figure S1). N-terminal peptides of each p53 proteoform were constructed using the N-terminal fragments absent in the N-terminal truncated proteoforms but present in the sequences of the longer proteoforms (Table S3). The success of protein expression was determined by probing the IVT expression by WB and luminescence assays using an anti-HaloTag monoclonal antibody that recognizes the HaloTag at the C-terminal or N-terminal end of the fusion proteoforms (Figure 3A,B) and an anti-p53 or p63 monoclonal antibody that specifically recognizes a peptide at the N-terminal end (amino acids 11–25 for p53 and 15–151 for p63), which is not present in the ΔN proteoforms of p53 (Figure 3C,D).

### 3.2. Evaluation of the Seroreactivity Potential of p53 and p63 Proteoforms in Colorectal Cancer Patients

Next, we proceeded to investigate the seroreactivity of the different p53 and p63 proteoforms and specific peptides, to determine the presence of cryptic epitopes in those polypeptides appearing from the alternative splicing, in order to determine whether a specific differential seroreactivity could exist among them, and to analyze for any improvement in their diagnostic ability in comparison to canonical proteins. To this end, we analyzed a total of 110 individual human plasma samples from CRC patients (*n* = 31, stages I–IV), colorectal premalignant individuals (*n* = 31), and healthy individuals (*n* = 48) (Table 1).

**Figure 4:**
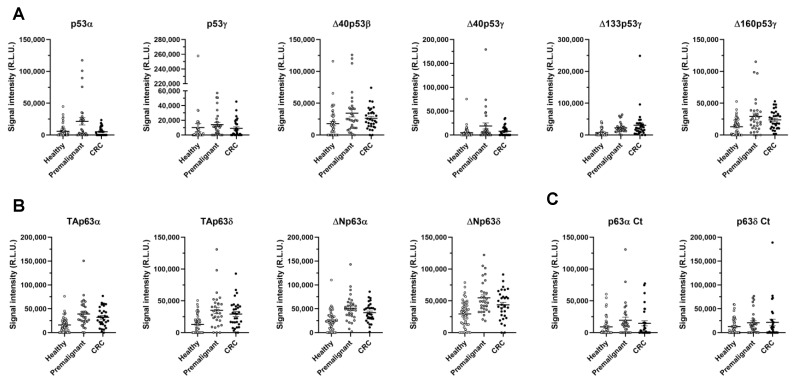
Evaluation of the seroreactivity levels of the p53 and p63 proteoforms and indicated specific cryptic peptides derived from the alternative splicing of *TP53* and *TP63* genes. (**A**) Significant CRC autoantibody levels against the indicated p53 proteoforms comparing healthy individuals, premalignant colorectal individuals, and CRC patients. (**B**) Significant CRC autoantibody levels against the indicated p63 proteoforms comparing healthy individuals, premalignant colorectal individuals, and CRC patients. (**C**) Autoantibody levels against the specific cryptic peptides of p63α and p63δ.

Seroreactivity to p53- and p63-derived proteoforms showed significant differences for most of the proteoforms tested in comparison with the control, the CRC, and the premalignant individuals’ plasmas, according to the threshold (cut-off value) obtained for all comparisons (Table 2). A statistically significant higher plasma seroreactivity, >1.4-fold and up to 4.2-fold for Δ133p53γ, was found in individuals with premalignant lesions or CRC patients, respectively, in comparison with healthy individuals for p53γ, Δ40p53β, Δ40p53γ, Δ133p53γ, and Δ160p53γ p53 proteoforms, and for TAp63α, TAp63δ, ΔNp63α, and ΔNp63δ p63 proteoforms (Table 2). Among these, only p53γ and Δ40p53γ showed significance when comparing healthy vs. premalignant individuals. In addition, the nine seroreactive proteoforms were able to discriminate CRC patients and premalignant individuals (pathological individuals) from healthy individuals, with plasma seroreactivity > 1.7-fold times for all of them except p53γ (Table 2 and Figure 4A,B).

Then, we focused on the identification of cryptic epitopes in p53 and p63 proteoforms appearing because of the different splicing. No differential seroreactivity was obtained for any p53-proteoform-specific peptide. However, p63α Ct and p63δ Ct showed higher but not significant seroreactivity (*p*-value < 0.1 and >1.7-fold) using plasma from individuals with premalignant lesions, in comparison with healthy individuals (Table 2). As only α and δ p63 peptides showed differential non-significant seroreactivity in individuals with premalignant lesions, these results suggest that the seroreactivity against these α and δ p63 proteoforms was associated not only with the differential C-terminal end peptide, but also with the conformational differences among proteoforms. In contrast, only p53γ proteoforms and Δ40p53β protein showed higher seroreactivity in individuals with premalignant lesions and CRC patients compared with healthy individuals. As the specific p53γ and p53β peptides were not differentially seroreactive to patients, p53 proteoforms might be conformational autoantigens of CRC patients.

**Figure 5:**
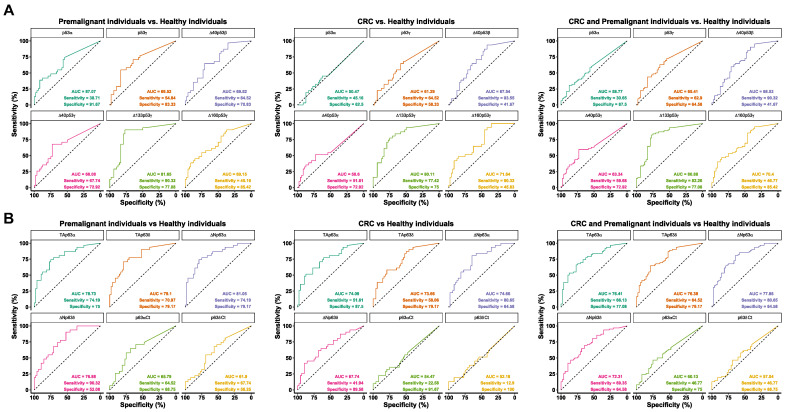
Diagnostic potential of the p53 and p63 proteoforms’ autoantibodies. The diagnostic value of autoantibodies against seroreactive p53 (**A**) and p63 (**B**) proteoforms was evaluated by means of ROC curves to discriminate between CRC patients, colorectal premalignant individuals, pathological individuals (CRC patients and premalignant lesion patients), and healthy individuals. AUC, sensitivity, and specificity for indicated comparisons are depicted.

We next analyzed the diagnostic ability of the different proteoforms, individually and in combination, by means of receiver operating characteristic (ROC) curves. Differential seroreactivity of p53 and p63 proteoforms showed individual areas under the curve (AUCs) to discriminate the pathological group (CRC patients and premalignant individuals) from healthy individuals up to 80.9% and 77.7%, with specificity up to 85.4% and 79.2%, and sensitivity up to 90.3% and 80.6%, respectively (Table S5 and Figure 5). Regarding the discrimination between CRC patients from healthy individuals, the differential seroreactivity to p53 and p63 proteoforms showed individual AUCs up to 80.1% and 74.7%, with specificity up to 75% and 89.6%, and sensitivity up to 93.55% and 80.6%. To discriminate premalignant individuals from healthy individuals, values of AUC were up to 81.6% and 81%, specificity up to 85.4% and 79.2%, and sensitivity up to 90.3% and 90.3%, respectively (Table S5 and Figure 5).

**Figure 6:**
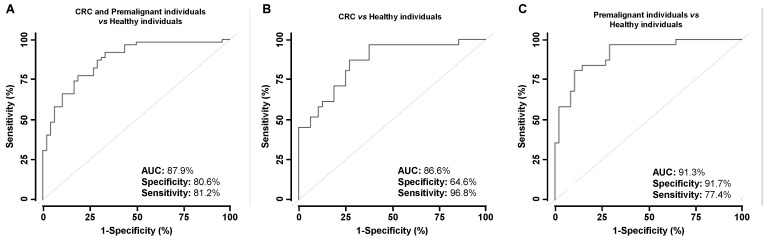
Evaluation of the diagnostic value of CRC and colorectal premalignant autoantibodies. The combination of the significant p53 and p63 proteoforms autoantibodies to discriminate (**A**) CRC and premalignant individuals from healthy individuals, (**B**) CRC from healthy individuals, and (**C**) premalignant individuals from healthy individuals showed AUCs higher than 85%, and sensitivity and specificity up to 96.8% and 87.5%, respectively.

Next, we investigated whether combined use of significant seroreactive p53 and p63 proteoforms would improve their individual diagnostic ability. In combination, ROC curves showed AUCs of 87.9%, 96.8%, and 91.3%, specificity of 80.6%, 64.6%, and 91.7%, and sensitivity of 77.4%, 81.2%, and 77.4% to discriminate CRC patients and premalignant individuals from healthy individuals, CRC patients from healthy individuals, and premalignant from healthy individuals, respectively (Figure 6).

Collectively, these results suggest that the measurement of p53 and p63 proteoforms increases the diagnostic potential of the individual measurement of p53 or p63 canonical proteins.

### 3.3. Analysis of the Diagnostic Value of the p53 and p63 Proteoforms as Targets of CRC Autoantibodies in POC-like Devices

**Figure 7:**
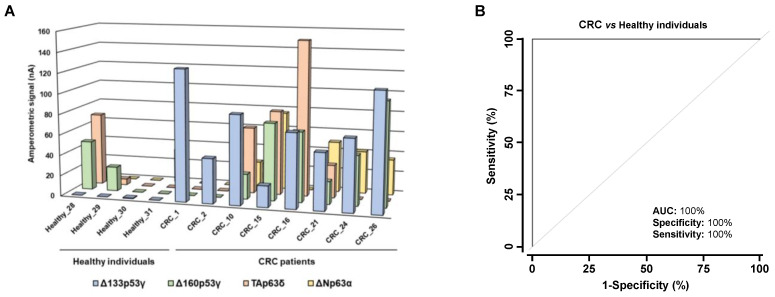
Autoantibody measurement in plasma samples by the proteoform-multiplexed electrochemical biosensing platform. (**A**) Amperometric responses obtained for the indicated proteoforms were larger for the CRC patients in comparison with the healthy individuals. (**B**) ROC curve of autoantibody detection against all proteoforms using the electrochemical biosensing platform. CRC, colorectal cancer patients.

Under previously optimized conditions [30,36,37], using human plasma, we next established a biosensing platform able to detect the presence of CRC autoantibodies against different proteoforms. The seroreactivity of random plasma samples from four controls and eight CRC patients at different stages against two p53 and two p63 differential seroreactive isoforms (Δ133p53γ, Δ160p53γ, TAp63δ and ΔNp63α) was analyzed as a proof of concept to establish a biosensing platform able simultaneously to measure different proteoforms for CRC detection. The control individuals showed lower seroreactivity in comparison with CRC patients, especially for the Δ133p53γ and ΔNp63α proteoforms (Figure 7A). Although significant results were obtained only for Δ133p53γ (*p*-value = 0.008), due to the number of samples analyzed with the biosensing platform, the detection of autoantibodies against these four p53 and p63 isoforms using electrochemical biosensors showed a high diagnostic ability for CRC, with AUC, sensitivity, and specificity of 100% (Figure 7B). The ideal parameters resulting from the analysis of ROC curves applying this type of device, even in a small cohort, already indicated in previous research, can be attributed to the extensive optimizations made in the development of these electrochemical bioplatforms [36,37,38], providing much better sensitivity and fewer false positives than conventional methodologies. We believe that the proof of concept demonstrated with this type of devices, competitive in terms of simplicity, affordability, and applicability at the point of care using conventional or state-of-the-art methodologies, gives significant added value to this work, in the context of the tremendous contemporary interest in advancing research and the implementation of egalitarian and sustainable precision medicine.

Collectively, our results suggest that the humoral immune response in cancer patients is highly complex against the p53 family of proteins. Indeed, the proteoforms derived from the alternative splicing of p53 and p63 possess different seroreactivity and for some specific proteoforms a higher diagnostic ability than the canonical proteins, suggesting that more than one proteoform of each member of the p53 family should be included in diagnostic platforms for the blood-based diagnosis of CRC patients.

## 4. Discussion

Cancer detection at early stages is the main approach to reduce cancer-related deaths, as treatments are more effective in the initial stages of the disease. Nowadays, more than 63% of colorectal cancer patients are diagnosed at III and IV late stages, when CRC cells have already metastasized, surgery is insufficient as treatment for the disease, and the 5-year survival rate is lower than 50%. In recent years, interest has increased in circulating biomarkers associated with CRC, such as circulant tumor cells (CTC), circulant tumor DNA (ctDNA), proteins, exosomes, or autoantibodies, as their measurement in blood is quick, minimally invasive, and painless. Detected by liquid biopsy, these blood-based biomarkers could be used as diagnostic or prognostic biomarkers, as well as markers of response to treatment, to improve the quality of life of patients and address the progression of the disease [39,40]. Because these cancer biomarkers can be released by cancer cells or surrounding cells (tumoral microenvironment, TME), different studies have focused on tissues and plasmas from patients and on cellular models for the identification of circulating biomarkers.

The humoral immune response associated with cancer has become an attractive tool for the early diagnosis of the disease [41,42,43]. Autoantibodies produced against specific tumor-associated autoantigens (TAAs, self-proteins altered during the progression of the disease by mutation, overexpression, splicing, frameshift, etc.) can be detected in plasma or serum samples and possess many advantages for diagnosis: (i) high stability, (ii) high specificity, (iii) easy-to-standardize seroreactivity assays, (iv) higher concentration in blood than other altered proteins, and (v) production months before the first clinical symptoms appear. Thus, they could be useful as early diagnostic biomarkers of the disease [28,44]. The mechanisms that lead to the production of specific autoantibodies remain unknown. However, theories include loss of tolerance, inflammation, changes in antigen expression and presentation patterns, reduced degradation, aberrant location, or altered structure [45].

In recent years, various approaches have been undertaken for the identification of CRC-specific TAAs and the characterization of their differential seroreactivity in plasma from CRC patients, such as immunoprecipitation coupled with mass spectrometry, protein microarrays, SEREX (serological analysis of recombinant cDNA expression libraries), or SERPA (serological proteome analysis) [32,33,37,46,47,48]. However, when using these techniques focusing only on canonical sequences, novel potential disease-specific autoantigens might be missed, such as protein proteoforms resulting from transcriptional regulation [32].

Here, we have characterized the humoral immune response in CRC patients against the different p53 and p63 proteoforms, as the p53 protein family has been widely associated with cancer and reported to regulate several biological processes, such as cell differentiation, proliferation, and apoptosis. These proteoforms possess both pro-tumoral and pro-metastatic potential and are involved in tumor progression and metastasis [49]. Only p53 mutants have been described to affect cancer progression, by inducing migration and invasion and altering the metastatic behavior of tumoral cells. However, the role of the different p53 proteoforms in cancer has not been elucidated [49,50,51]. Regarding p63, both TAp63 and ΔNp63 proteoforms have been associated with cancer development and progression and have been described as metastatic inhibitors. Meanwhile, TAp63 proteoforms are related to cell apoptosis and senescence, and ΔNp63 proteoforms promote cellular survival and proliferation, and have been described as inhibitors of p53 proteins [22,52]. However, better understanding is still required of the roles of p53 and p63 proteoforms in tumorigenesis, cancer progression, and metastasis. Because the protein expression levels of p53 and p63 proteoforms are tightly regulated during cancer formation and progression, they can trigger a specific humoral immune response, which can be useful for blood-based diagnosis of CRC. In this study, p73 proteoforms were not included as their potential as autoantigens associated with CRC has previously been described [32]. In addition, ε p63 proteoforms were not included either, as they only differ from the canonical protein in an 85 amino acid sequence missing at the C-terminal end of ε p63 proteoforms [52].

Among p53 proteoforms, p53γ, Δ40p53β, Δ40p53γ, Δ133p53γ, and Δ160p53γ showed significantly higher differential seroreactivity in CRC patients and colorectal premalignant individuals compared with healthy individuals. Specifically, autoantibodies against all of them were especially able to discriminate individuals with premalignant lesions from healthy individuals (AUC about 70%), and all of them except p53γ and Δ40p53γ discriminated CRC patients from healthy individuals (AUC higher than 70%). Furthermore, Δ133p53γ showed the highest diagnostic capacity for CRC patients and individuals with premalignant lesions, which correlates with previous findings showing that this protein is highly dysregulated in CRC and possesses a key role in its development and progression, favoring resistance processes while promoting cell growth, invasion, and metastasis [26,53], and thus suggesting the specific induction of an humoral immune response to this proteoform in CRC patients. The p63 proteoforms TAp63α, TAp63δ, ΔNp63α, and ΔNp63δ showed statistically significant higher seroreactivity in patients compared with healthy individuals. Autoantibodies against the four proteins were able to discriminate premalignant individuals from healthy individuals with an AUC close to 80%, and CRC patients from healthy individuals with an AUC close to 70%. In addition, we analyzed the differential seroreactivity against specific N-terminal and C-terminal end peptides of each proteoform to elucidate whether any of them contained cryptic epitopes of autoantibodies. p53-specific peptides did not show differential seroreactivity in patients, suggesting that these seroreactive proteoforms contain mostly conformational epitopes. In addition, tridimensional structure prediction for p53 proteoforms showed that p53γ proteoforms might possess less compact structures and major unfolding regions, which could trigger the humoral immune response. In contrast, α and δ p63 peptides were more seroreactive against individuals with premalignant lesions in comparison to healthy individuals, suggesting that these C-terminal peptides should contain linear epitopes that trigger the humoral immune response in patients. However, as the differential seroreactivity of these peptides per se was lower than that of the full-length α and δ p63 proteoforms, other conformational epitopes contained in their structures might be involved in the higher seroreactivity against these proteins. The differential seroreactivity was assessed by the luminescence HaloTag-bead assays as well as by the electrochemical biosensing strategy, which confirmed by two different approaches the diagnostic ability of specific p53 and p63 proteoforms in CRC.

Interestingly, canonical p53 has been previously described as an autoantigen in cancer, but with a low sensitivity (lower than 25%) [54]. Although autoantibodies against p53α were detected in the HaloTag-based luminescence assays performed in this study, its seroreactivity in CRC patients did not differ greatly from healthy individuals, in contrast to other p53 and p63 proteoforms that were able to discriminate CRC patients from healthy individuals, with high sensitivity and specificity. In addition, although higher seroreactivity against p53α was observed in individuals with premalignant lesions in comparison with healthy individuals, the selected seroreactive p53 and p63 proteoforms showed higher diagnostic ability in individuals with premalignant lesions. Thus, the combination in autoantigen panels of p53α with the other proteoforms here demonstrated with differential seroreactivity might help to improve the diagnosis of CRC, increasing the sensitivity and specificity of the panel of autoantigens. In addition, the integration into POC devices of multiple autoantigens specific to CRC might be useful for routine clinical diagnosis.

Finally, the seroreactivity assays performed in this study confirmed the high ability of the most seroreactive p53 and p63 proteoforms to discriminate CRC patients and individuals with premalignant lesions from healthy individuals, in comparison with canonical proteins. In these seroreactivity assays, HaloTag fusion proteins were immobilized into the MBs in an oriented position and conformation, allowing better recognition of the IgGs from patients, thus requiring low volumes of plasma samples and HaloTag fusion proteins. However, to overcome the limitations of the current study, validation with a larger cohort of samples from different hospitals should also be performed for further demonstration of the diagnostic value of the proteoforms of the p53 family, not only in CRC but also in other cancer types. Additionally, samples from other cancer types should also be analyzed to determine whether the presence of a specific humoral immune response to p53 and p63 proteoforms is specific to CRC, or would also be observed in other major solid tumors, and whether some proteoforms are also associated with different cancer types. As we envision the use of the markers described here for primary screening of CRC, these limitations should be addressed prior to their integration with other CRC markers, in order to obtain the adequate specificity and sensitivity to detect colorectal premalignant individuals and CRC patients among an analyzed population by means of a simple blood test.

## 5. Conclusions

We here show that the p53 and p63 proteoforms with substantial differences in their primary sequences and their 3D structure in comparison with the canonical proteins possess, not only differential seroreactivity but also, for some proteoforms, a higher diagnostic ability to distinguish CRC patients and colorectal premalignant individuals from healthy individuals. Additionally, our results suggest that proteoforms derived from alternative splicing of the autoantigens most relevant in cancer should be evaluated to determine their usefulness for increasing the diagnostic ability of the canonical proteins. Finally, we developed POC-like devices for CRC detection with selected proteoforms, capable of clinical implementation, that could be integrated with previously described CRC-specific TAAs for blood-based early diagnosis of the disease [30,32,33,35,37].

## Figures and Tables

**Table 1:** Information of plasma samples used for the seroreactivity assays from healthy individuals, individuals with premalignant lesions, and CRC patients.

	Sample	Number (*n*)	Age Average ± SD (Years)	Age Range (Years)	Gender (*n*)		CRC Stage (*n*)			
					Male	Female	I	II	III	IV
*Luminescence assays*	Healthy individuals	48	67.08 ± 17.60	36–101	23	25	-			
	Premalignant lesions	31	63.77 ± 6.50	54–79	21	10	-			
	CRC patients	31	70.90 ± 13.95	38–86	15	16	3	2	15	11
*Electrochemical biosensing*	Healthy individuals	4	67.50 ± 6.45	63–74	2	2	-			
	Premalignant lesions	4	65.25 ± 1.71	63–67	2	2	-			
	CRC patients	8	71.00 ± 11.70	59–86	1	7	2	-	3	3

**Table 2:** Data analysis of p53 and p63 proteoform luminiscence seroreactivity assays.

	Healthy		Premalignant		CRC		Pathology		Threshold ^			*p*-Value		
Proteoform	Mean	SEM	Mean	SEM	Mean	SEM	Mean	SEM	(H vs. Pre) *	(H vs. CRC) *	(H vs. P) *	(H vs. Pre) *	(H vs. CRC) *	(H vs. P) *
p53α	5793	1433	21,387	5721	5135	1255	13,261	3085	22,773	3361	13,280	0.0080	0.9432	0.0980
p53β	16,312	4724	17,134	5269	12,151	3947	14,642	3280	23,059	4743	47,192	0.9025	0.8287	0.8359
p53γ	10,090	5399	14,142	2986	9113	2072	11,628	1831	10,016	1396	2383	0.0026	0.0784	0.0045
Δ40p53α	20,732	5262	12,965	8135	10,503	3186	11,734	4335	12,547	314	12,547	0.0613	0.5312	0.1342
Δ40p53β	17,802	3195	34,370	5965	26,365	2994	30,368	3349	21,224	6920	6920	0.0034	0.0084	0.0009
Δ40p53γ	4960	1687	18,959	6372	7982	1829	13,470	3361	3289	3152	3152	0.0053	0.1745	0.0131
Δ133p53α	27,121	13,961	17,050	3921	15,271	3010	16,160	2454	20,620	10,045	20,620	0.5712	0.5765	0.4984
Δ133p53β	18,875	3200	20,586	3906	20,598	3608	20,592	2637	5794	10,265	5794	0.7425	0.6167	0.6164
Δ133p53γ	7295	1616	22,708	3020	30,647	8125	26,678	4328	9474	8230	9474	0.0000	0.0000	0.0000
Δ160p53α	41,082	8238	28,720	3545	27,919	4281	28,319	2757	8925	18,463	18,167	0.8327	0.8326	1.0000
Δ160p53β	40,157	6085	31,251	5123	34,769	6249	33,010	4013	33,587	65,132	33,587	0.4304	0.7823	0.5207
Δ160p53γ	13,117	1870	29,256	5160	23,874	2577	26,565	2881	26,147	7124	26,147	0.0041	0.0012	0.0003
TAp63α	16,148	2226	39,160	5211	32,113	3633	35,637	3182	23,630	30,240	24,128	0.0000	0.0003	0.0000
TAp63β	28,778	4809	17,873	4547	35,166	12,679	26,520	6771	26,860	16,164	28,653	0.1680	0.9878	0.4050
TAp63γ	31,057	6352	37,917	8951	34,715	10,443	36,316	6824	35,915	38,003	31,196	0.7591	0.9069	0.7972
TAp63δ	12,998	2064	35,005	4944	29,176	3879	32,091	3138	22,093	21,769	21,769	0.0000	0.0004	0.0000
ΔNp63α	24,323	3057	51,002	4959	41,914	3554	46,458	3081	36,392	27,973	27,973	0.0000	0.0002	0.0000
ΔNp63β	20,404	4843	27,926	7202	20,176	4397	24,051	4214	2135	37,008	15,429	0.7190	0.5817	0.5823
ΔNp63γ	23,568	4232	34,393	12,519	32,923	6528	33,658	7002	13,056	8122	7230	0.9143	0.1754	0.3773
ΔNp63δ	29,457	2959	54,744	4673	43,705	4122	49,225	3170	32,007	52,889	37,134	0.0001	0.0081	0.0001
p53Nt	29,293	3665	35,447	9143	40,625	10,808	38,036	7028	33,231	12,771	32,674	0.4847	0.7745	0.8069
Δ40p53Nt	47,605	9055	49,939	17,142	30,812	4116	40,376	8828	30,280	38,814	38,847	0.5943	0.2497	0.3108
Δ133p53Nt	55,418	7128	46,807	7534	52,115	10,629	49,461	6469	32,579	64,396	64,396	0.6192	0.8803	0.6952
Δ160p53Nt	85,145	18,721	53,696	6032	47,763	4415	50,729	3726	81,010	76,414	81,010	0.6192	0.4218	0.4333
p53αCt	6277	1710	10,212	2900	4125	1944	7169	1774	13,882	42,926	12,780	0.6080	0.1955	0.6544
p53βCt	5645	1870	5098	1741	12,408	7642	8753	3915	15,463	82,634	15,463	0.8585	0.3421	0.4952
p53γCt	11,121	3310	17,343	3987	9852	3586	13,598	2702	7471	38,470	4373	0.1598	0.5660	0.6056
TAp63Nt	28,242	5042	33,052	6496	29,976	5025	31,514	4077	24,042	8922	23,957	0.5538	0.3720	0.3720
ΔNp63Nt	33,492	5719	31,462	6993	46,664	10,231	39,063	6222	14,546	38,591	34,511	0.7488	0.5865	0.8955
p63αCt	9109	2140	19,383	4923	14,656	4140	17,019	3204	8884	30,323	10,516	0.0151	0.4862	0.0608
p63βCt	20,646	6678	17,065	2935	18,355	4458	17,710	2648	12,826	233	24,377	0.3322	0.6048	0.7836
p63γCt	10,515	1793	12,553	3423	13,825	3720	13,189	2508	4896	934	4896	0.9629	0.7655	0.8339
p63δCt	12,734	2402	20,809	4205	21,350	6924	21,079	4017	7405	65,040	13,241	0.0717	0.7403	0.1990

* H, healthy. Pre, premalignant. CRC, colorectal cancer. P, pathological group (premalignant individuals and CRC patients). ^, threshold value for the discrimination between indicated comparisons.

## Data Availability

The data presented in this study are available in the main body of the manuscript and in the supplementary information.

## Supplementary Materials

The following supporting information can be downloaded at: https://www.mdpi.com/article/10.3390/cancers15072102/s1, Figure S1: Confirmation of the correct cloning of the p53 and p63 full length proteoforms and specific N-terminal and C-terminal end peptides into the pDONR221 donor vector and the pANT7_cHalo or pJFT7_nHalo expression vectors was assessed by agarose gel electrophoresis of plasmids isolated from *E. coli* cells after transformation; Figure S2: The original western blot figures of Figure 3; Table S1: Information of the plasma samples used in the study from healthy individuals (Healthy), individuals with premalignant lesions (PRE), and CRC patients (CRC); Table S2: Oligonucleotides used for amplification of p53 and p63 proteoforms from splicing and specific N-terminal and C-terminal peptides; Table S3: Molecular data of full-length proteoforms, specific peptides, and Halo protein cloning for seroreactivity analysis by number of base pairs (bp), number of amino acids (aa), and molecular weight (kDa); Table S4: List of antibodies used for WB, luminescence, and biosensing assays; Table S5: Specificity, sensitivity, and area under the curve (AUC) obtained from ROC curve analysis for the determination of the diagnostic ability of specific autoantibodies.
