# Supplementary material for: p53 and p63 Proteoforms Derived from Alternative Splicing Possess Differential Seroreactivity in Colorectal Cancer with Distinct Diagnostic Ability from the Canonical Proteins
Source: Cancers (Basel). 2023 Mar 31;15(7):2102. doi: 10.3390/cancers15072102 (PMC10092954; doi:10.3390/cancers15072102)
Supplement: Supplementary file 1 [file cancers-15-02102-s001.zip › Supplementary Figure S1.pdf]

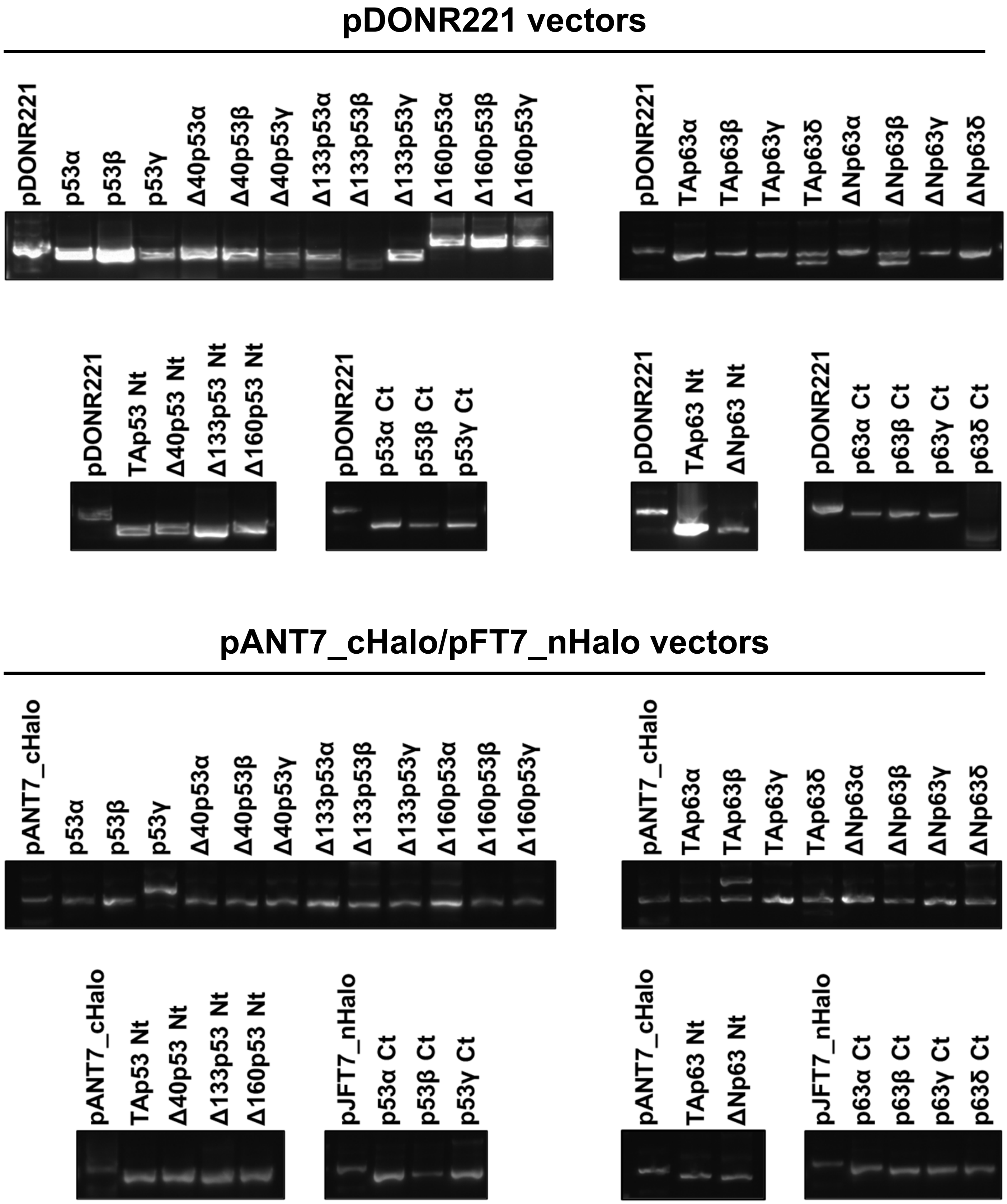

Supplementary Figure S1. Confirmation of the correct cloning of the p53 and p63 full length proteoforms and specific N-terminal and C-terminal end peptides into the pDONR221 donor vector and the pANT7_cHalo or pJFT7_nHalo expression vectors was assessed by agarose gel electrophoresis of plasmids isolated from *E. coli* cells after transformation.
