# Supplementary figures and images for: p53 and p63 Proteoforms Derived from Alternative Splicing Possess Differential Seroreactivity in Colorectal Cancer with Distinct Diagnostic Ability from the Canonical Proteins
Source: Cancers (Basel). 2023 Mar 31;15(7):2102. doi: 10.3390/cancers15072102 (PMC10092954; doi:10.3390/cancers15072102)

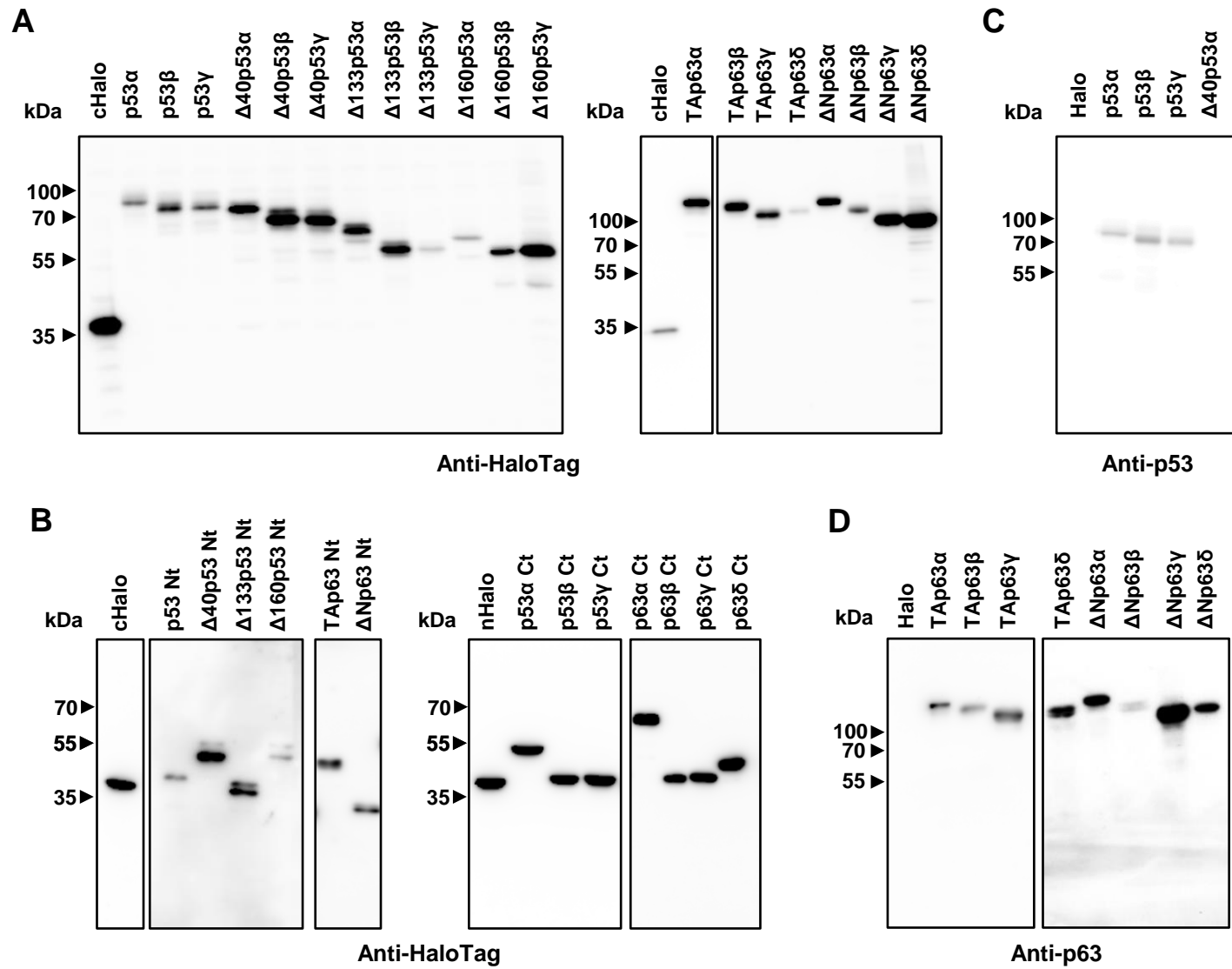

**Supplementary Figure S2.** The original western blot figures of Figure 3.

Supplement: Supplementary file 1 [file cancers-15-02102-s001.zip › Supplementary Figure S2.pdf]
